# Change in mammography screening attendance after removing the out-of-pocket fee: a population-based study in Sweden (2014–2018)

**DOI:** 10.1007/s10552-021-01476-4

**Published:** 2021-07-28

**Authors:** Magdalena Lagerlund, Anna Åkesson, Sophia Zackrisson

**Affiliations:** 1 Department of Translational Medicine, Diagnostic Radiology, Skåne University Hospital, Lund University, Malmö, Sweden; 2 Clinical Studies Sweden – Forum South, Skåne University Hospital, Lund, Sweden

**Keywords:** Mammography, Breast cancer screening, Women’s health, Socioeconomic aspects of health

## Abstract

**Purpose:**

To assess the change in mammography screening attendance in Sweden—overall and in sociodemographic groups at risk of low attendance—after removal of the out-of-pocket fee in 2016.

**Methods:**

Individual-level data on all screening invitations and attendance between 2014 and 2018 were linked to sociodemographic data from Statistics Sweden. Odds ratios and 95% confidence intervals (CIs) for attendance by time period and sociodemographic factor were computed using mixed logistic regression to account for repeated measures within women. The study sample included 1.4 million women, aged 40–75, who had a mammography screening appointment in 2014–2015 and/or 2017–2018 in 14 of Sweden’s 21 health care regions.

**Results:**

Overall screening attendance was 83.8% in 2014–2015 and 84.1% in 2017–2018 (+ 0.3 percentage points, 95% CI 0.2–0.4). The greatest increase in attendance was observed in non-Nordic women with the lowest income, where attendance rose from 62.9 to 65.8% (+ 2.9 points, 95% CI 2.3–3.6), and among women with four or more risk factors for low attendance, where attendance rose from 59.2 to 62.0% (+ 2.8 points, 95% CI 2.2–3.4).

**Conclusion:**

Screening attendance did not undergo any important increase after implementing free screening, although attendance among some sociodemographic groups increased by almost three percentage points after the policy change.

## Introduction

Most European countries have national population-based programs that offer mammography screening to women in varying age ranges between 40 and 74 [1]. Since the public health impact of population-based screening depends on high attendance in order to reduce breast cancer mortality, monitoring, as well as understanding and considering the factors influencing attendance, is important. In the European guidelines for quality assurance in breast cancer screening and diagnosis, attendance is listed as one of the key performance indicators where > 70% is stated as the acceptable level and > 75% as the desirable level [2].

Sweden has offered a nationwide outreach mammography screening program since 1997 [3, 4]. Overall attendance is typically about 80% [1], but is lower in groups who may be socioeconomically vulnerable, such as women who were born abroad [5–8], have a low income [6–8], are unmarried or living without a partner [5, 7–10], are not gainfully employed [5–7, 10], or who have a lower education [5].

As part of the Swedish government’s efforts to improve health care equality and women’s health, mammography screening became free of charge on July 1 2016 [11], before which time most regions charged a small out-of-pocket fee (≤ 200 SEK ≈ $23 USD). According to research conducted in the USA, reducing or removing the out-of-pocket fee can increase screening attendance [12–14]. However, a Swedish study found no correlation between fees (0–170 SEK ≈ $0–20 USD) and attendance (66–91%) in different regions in Sweden in 1995–1996 [4]. In a more recent study conducted in Stockholm County (Sweden), attendance rose from 68 to 70% after the screening fee was removed in 2012 [15].

The objective of this study was to assess the change in mammography screening attendance in Sweden—overall and in sociodemographic groups at risk of low attendance—after removal of the out-of-pocket screening fee in 2016. We examined the change in attendance from 2014–2015 to 2017–2018, stratified by region and sociodemographic factors.

## Materials and methods

This longitudinal population-based register study was conducted in Sweden, where women between the ages of 40 and 74 are invited to mammography screening every 18–24 months depending on age and regional capacity. All invitations are sent by post and offer a pre-booked appointment date and time, which does not need to be confirmed and can be rescheduled or canceled. Since each health care region individually conducts and administers their screening, there are differences in intervals between screening appointments, the layout and content of the invitation letter, hours of operation, ways of canceling or rescheduling appointments, reminders, etc. All but two (Stockholm and Östergötland) of the 21 health care regions charged a small out-of-pocket fee between 80 and 200 SEK (≈$9–23 USD) before the implementation of free screening in 2016.

A study period between 2014 and 2018 was chosen to study the change in screening attendance during the two-year period before and after removal of the out-of-pocket fee in 2016. Individual screening-related data were extracted for all women invited to the screening program in 15 of 21 health care regions in Sweden. These regions used the same company (Sectra AB) for their radiological information system (RIS) to administer and track invitations, attendance, and results throughout the entire study period, which enabled high quality and consistency of data between regions. Of the six regions initially excluded, four (Jönköping, Kronoberg, Norrbotten, and Uppsala) used different radiological information systems for all or part of the study period, and two (Sörmland and Östergötland) did not grant us permission to extract data. Two of three programs operating in the Stockholm region granted permission. However, these were excluded from the final study sample, since they had already removed the out-of-pocket fee before the study period. This study was approved by the local ethics committee at Lund University (Nos. 2018/576 and 2018/965). Active informed consent as a requirement for data collection was waived.

The extracted data included the screening appointment date, age at the screening appointment, and attendance outcome (attended, canceled, missed, and unavailable), for each regional mammography program separately, and were combined into one dataset. The unique personal identity number assigned to every resident in Sweden was used to merge screening data with information on individual-level sociodemographic characteristics obtained from population registers at Statistics Sweden (the Longitudinal Integration Database for Health Insurance and Labour Market Studies (LISA) [16], the Total Population Register [17], and the Geodatabase [18]). To secure anonymity, Statistics Sweden replaced this number with an arbitrary code before releasing the data to the research group. The most recent sociodemographic information was used for each screening appointment. Same-year sociodemographic data were linked to each screening appointment in 2014, 2015, and 2017; in 2018, same-year data were available only for home ownership and type of municipality, and data on income, education, and cohabitation from 2017 were used.

**Fig. 1:**
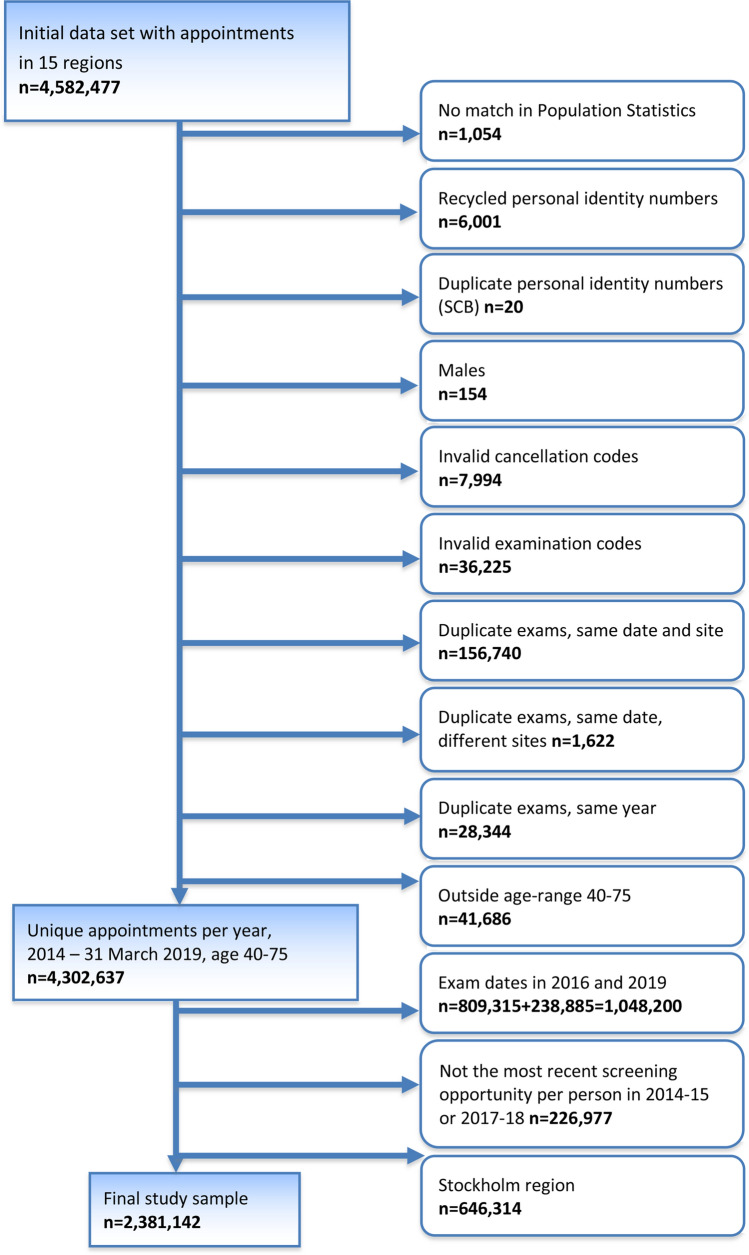
Selection of the final study sample

**Fig. 2:**
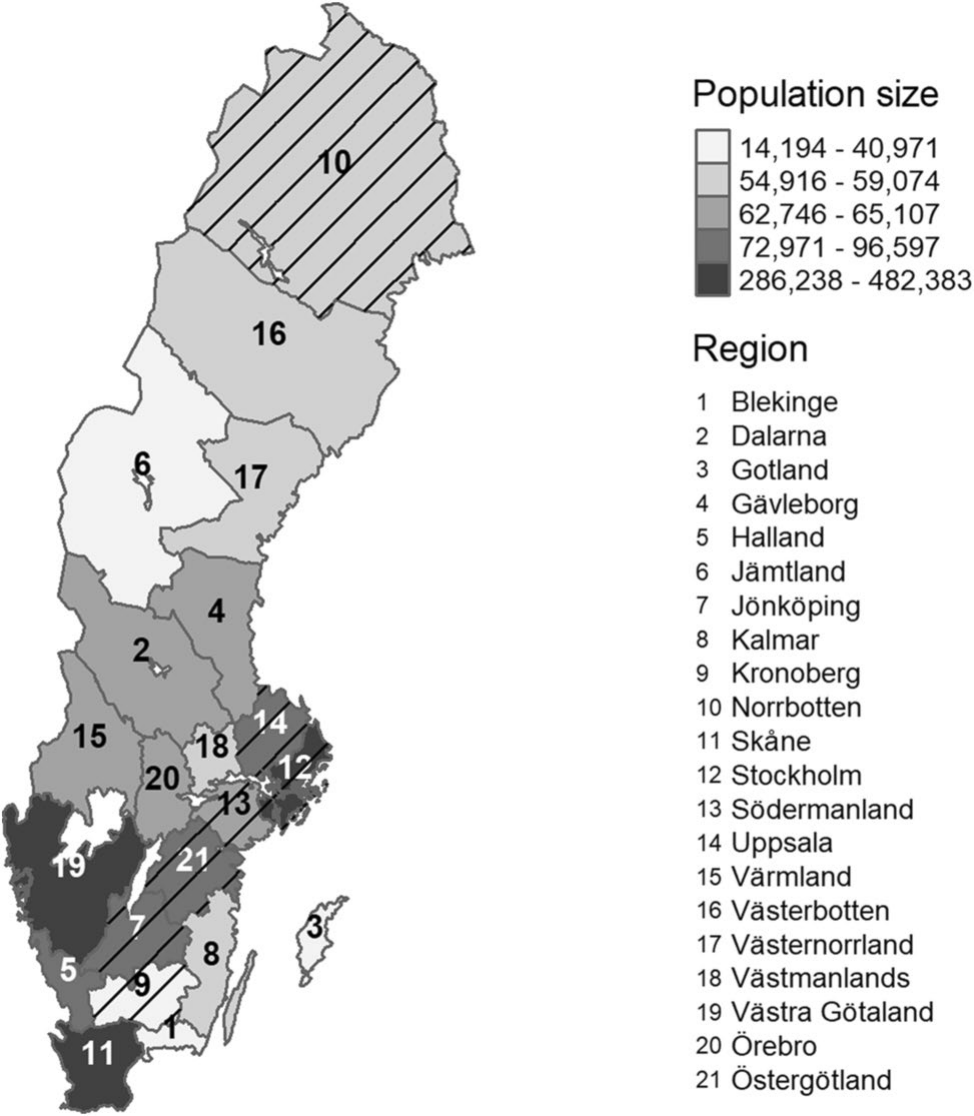
Map of the Swedish health care regions showing the population of women aged 40–75 in 2018. Excluded regions are hatched

Initially, the dataset included a total of 4,582,477 appointments among 1,780,164 women in 15 regions (including Stockholm). The flow chart in Fig. 1 describes the different steps of exclusion, resulting in a final selection of 2,381,142 appointments among 1,350,654 women in 14 regions (Fig. 2). These 14 regions encompass about 59% of the women eligible for mammography screening in Sweden and about 81% of the women affected by the fee removal. The most recent screening appointment for each woman was selected, aged 40–75, within each time period (2014–2015 and 2017–2018), excluding appointments during the transition year of 2016. Women 75 years of age were included to allow for overflow from the age limit of 74 years due to administrative reasons, e.g., rescheduling. Appointments were excluded when personal identity numbers lacked a match, had duplicates, or were suspected to have been recycled according to data from Statistics Sweden. Furthermore, appointments with examination or cancelation codes that were not related to mammography screening were excluded. Duplicate appointments within the same program (both identical and non-identical) at different locations and within the same year were excluded as well.

### Outcome variable

The outcome variable in this study was mammography screening attendance (yes/no), irrespective of whether it was the original or a rescheduled appointment date, according to the most recent screening appointment for each woman during the periods 2014–2015 and 2017–2018. The rationale for studying two-year time periods was to allow for longer screening cycles, which is common in several regions.

### Sociodemographic and program-related variables

Sociodemographic variables and categorizations are presented in Table 1 and include age group, cohabitation (in which only couples who have children together were categorized as cohabiting), level of education, income (individual share of equivalized disposable household income in SEK), main source of income, home ownership, country of birth, and type of municipality (based on categorization by the Swedish Association of Local Authorities and Regions) [19]. Program-related variables included region, out-of-pocket fee in 2015, and year of screening appointment. Variables were categorized based on the way in which they were provided by Statistics Sweden, and by logically and conceptually combining categories without losing important differences in the attendance outcome, in order to minimize the number of categories. Missing values, which were excluded from the analyses, ranged from none (region, year, and age) to 1.2% for education and 2.3% for home ownership in 2014–2015 and were below 0.5% for all other variables (Table 1). All variables were analyzed as categorical variables.

**Table 1:** Sociodemographic and other characteristics of the study sample at the time of the most recent mammography screening appointment in Sweden (2014–2015 and 2017–2018)

Characteristic	2014–2015 *N* = 1,191,609		2017–2018 *N* = 1,189,533		Standardized difference^a^
	*N*	%	*N*	%	%
Mean age (SD)	56.12	(10.06)	56.37	(10.10)	7.87
Age group (years)					
40–44	192,887	16.19	185,197	15.57	1.69
45–49	186,686	15.67	176,442	14.83	2.32
50–54	172,539	14.48	188,375	15.84	3.78
55–59	164,171	13.78	162,726	13.68	0.28
60–64	163,671	13.74	159,841	13.44	0.87
65–69	173,291	14.54	159,718	13.43	3.22
70–75	138,364	11.61	157,234	13.22	4.87
Cohabitation (living with partner)					
Yes	721,892	60.58	721,839	60.68	0.21
No	464,111	38.95	464,613	39.06	0.23
*Missing*	5,606	0.47	3,081	0.26	3.51
Level of education					
Low (elementary school, ≤ 9 years)	193,073	16.20	171,287	14.40	5.01
Intermediate (secondary school)	550,549	46.20	542,545	45.61	1.19
High (post-secondary)	433,252	36.36	462,339	38.87	5.18
*Missing*	14,735	1.24	13,362	1.12	1.05
Income category^b^					
Lowest (decile 1)	118,754	9.97	118,677	9.98	0.04
Low–medium (decile 2–4)	355,513	29.83	355,832	29.91	0.17
Medium–high (decile 5–10)	711,736	59.73	711,944	59.85	0.25
*Missing*	5,606	0.47	3,080	0.26	3.51
Main source of income					
Employment	693,208	58.17	716,433	60.23	4.18
Retirement pension	315,836	26.51	299,031	25.14	0.32
Student finance	4,574	0.38	4293	0.36	0.38
Care of a sick child or relative	5,777	0.48	6,090	0.51	0.39
Social assistance and benefits					
Sickness benefit	25,046	2.10	25,792	2.17	0.46
Sickness compensation	74,548	6.26	67,550	5.68	1.19
Unemployment insurance/benefit	7382	0.62	6298	0.53	2.44
Labour market program	15,125	1.27	14,261	1.20	0.64
Financial assistance	20,339	1.71	23,107	1.94	1.76
No income	24,168	2.03	23,598	1.98	0.32
*Missing*	5,606	0.47	3,080	0.26	3.51
Home ownership					
Yes (house or apartment)	883,642	74.16	882,378	74.18	1.63
No	280,078	23.50	287,870	24.20	0.05
*Missing*	27,889	2.34	19,285	1.62	5.16
Country of birth					
Sweden	1,001,904	84.08	979,249	82.32	4.70
Nordic country (except Sweden)	73,001	6.13	78,051	6.56	1.79
Europe (except Sweden and Nordic countries)	72,832	6.11	92,728	7.80	6.62
Other	43,813	3.68	39,427	3.31	1.97
*Missing*	59	0.00	78	0.01	0.21
Region					
Blekinge	32,735	2.75	33,110	2.78	0.22
Dalarna	63,497	5.33	60,682	5.10	1.02
Gotland	13,577	1.14	13,832	1.16	0.22
Gävleborg	62,516	5.25	60,775	5.11	0.62
Halland	64,814	5.44	65,874	5.54	0.43
Jämtland/Härjedalen	25,501	2.14	26,699	2.24	0.71
Kalmar	51,321	4.31	50,630	4.26	0.25
Skåne	265,192	22.25	271,723	22.84	1.41
Värmland	60,219	5.05	59,993	5.04	0.05
Västerbotten	50,257	4.22	49,076	4.13	0.46
Västernorrland	50,328	4.22	50,988	4.29	0.31
Västmanland	53,995	4.53	47,125	3.96	2.83
Västra Götaland	342,100	28.71	348,371	29.29	1.27
Örebro	55,557	4.66	50,655	4.26	1.96
Type of municipality					
Large cities (> 200,000)^c^	294,396	24.71	305,857	25.71	2.32
Mid-sized cities (50,000–200,000)^d^	455,161	38.20	451,125	37.92	0.56
Smaller cities, towns, and rural areas	436,446	36.63	429,897	36.14	1.01
*Missing*	5,606	0.47	2,654	0.22	4.21
Out-of-pocket fee (2015) in SEK^e^					
80	55,557	4.66	50,655	4.26	1.96
100	393,421	33.02	399,001	33.54	1.12
120	297,927	25.00	304,833	25.63	1.44
150	204,140	17.13	204,243	17.17	0.10
200	240,564	20.19	230,801	19.40	1.97
Year of scheduled appointment					
2014	542,148	45.50			
2015	649,461	54.50			
2017			542,602	45.61	
2018			646,931	54.39	

^a^The difference between the groups divided by the pooled standard deviation; a value greater than 10% is interpreted as a meaningful difference

^b^Income categories for 2014–15: Lowest: ≤ 92,700 SEK; low–medium: 92,800–150,400 SEK; medium–high: ≥ 150,500 SEK. Income categories for 2017–18: Lowest: ≤ 98,300 SEK; low–medium: 98,400–162,700 SEK; medium–high: ≥ 162,800 SEK

^c^Includes commuting zone

^d^Includes neighboring municipalities

^e^80 (Örebro), 100 (Kalmar and Västra Götaland), 120 (Blekinge and Skåne), 150 (Dalarna, Halland, Jämtland, and Västernorrland), 200 (Gotland, Gävleborg, Värmland, Västerbotten, and Västmanland)

### Statistical analysis

Standardized differences were calculated to examine change in the sociodemographic distribution of the study sample between time periods, with a value greater than 10% considered potentially meaningful. This measure describes the difference between the groups divided by the pooled standard deviation. The percentage of screening appointments attended was calculated for each time period, by region and sociodemographic factor, and reported with 95% confidence intervals (CIs). Change in attendance before and after removing the out-of-pocket fee was reported in percentage points with 95% CIs. We identified six large sociodemographic groups (*n* > 50,000) with attendance below 80% in 2014–2015 and further examined change in attendance before and after the fee removal in each of these groups and combinations thereof. Since the same women can be included in both time periods, mixed logistic regression was used to account for the correlation of observations within individuals. Results are presented as odds ratios (ORs) and 95% CIs for mammography attendance in 2017–2018 vs. 2014–2015. We calculated unadjusted estimates, as well as estimates adjusted for the potential confounding effect of various sociodemographic factors. Statistical software used for the analyses were SPSS, version 25, and R, version 4.0.

## Results

The study sample among the 14 included health care regions contained a total of 2,381,142 appointments among 1,350,654 women, with 1,191,609 appointments in 2014–2015 and 1,189,533 appointments in 2017–2018. A total of 1,032,810 women had an appointment in each time period.

Descriptive characteristics of the study sample in each two-year time period are presented in Table 1. In 2014–2015, the mean age at the time of the screening appointment was 56 years; 61% of women were living with a partner; 16% had a low level of education; 12% got their main income from social assistance and benefits and 85% from employment or retirement income; 74% owned their home; 84% were born in Sweden; 25% lived in a large city or surrounding commuting areas; and 37% lived in smaller cities and rural areas. Regions with the largest share of the sample were Västra Götaland (28.7%) and Skåne (22.3%). Most proportions were stable between time periods; all of the standardized differences were below 10%.

The overall attendance for all regions combined was 83.8% in 2014–2015 and 84.1% in 2017–2018 (+ 0.3 percentage points, 95% CI 0.2–0.4). Attendance per region and by sociodemographic factor are presented in Table 2. There was no change in attendance between 2014–2015 and 2017–2018 in the group of regions with the lowest fees (80–120 SEK) in 2015. A larger increase occurred in the regions that charged 150 SEK than among regions charging 200 SEK. In 2014–2015, Gotland had the lowest attendance (81.0%), followed by Örebro (82.0%) and the highest attendance was achieved in Jämtland/Härjedalen (86.1%), followed by Värmland (85.9%) and Västmanland (85.5%). A statistically significant increase in attendance from 2014–2015 to 2017–2018 occurred in seven regions, with the highest increase in Västernorrland (+ 3.1 points), Västerbotten (+ 1.4 points), and Halland (+ 1.3 points). In four regions, there was a statistically significant decrease in attendance, with the largest decrease in Örebro (− 1.3 points) and Blekinge (− 1.1 points). Attendance according to age groups varied from 82.2% among women in their forties to 86.0% among women in their sixties in 2014–2015. The largest increase over time was found among women 70–75 years of age (+ 1.0 points). In 2014–2015, attendance was least prevalent among women who lived without a partner (76.9%), who had a low level of education level (78.0%), who were in the lowest income decile (71.8%), whose main source of income was not employment or retirement pension (e.g., 68.8% among women with social assistance or benefits and 56.0% among those with no income), who did not own their home (73.5%), and who were not born in Sweden and especially among those born outside of the Nordic countries (70.8%).

**Table 2:** Mammography screening attendance with 95% confidence intervals (CI) and change in percentage points (PP) from 2014–2015 to 2017–2018 by sociodemographic factor (n = number of attenders)

Variable	2014–2015 (*N* = 1,191,609)				2017–2018 (*N* = 1,189,533)				Change (95% CI)		
	*n*	%	Lower CI	Upper CI	*n*	%	Lower CI	Upper CI	PP	Lower CI	Upper CI
Total	998,533	83.80	83.73	83.86	999,913	84.06	83.99	84.13	0.26	0.17	0.36
Age group											
40–44	158,851	82.35	82.18	82.52	153,410	82.84	82.66	83.01	0.48	0.24	0.72
45–49	153,046	81.98	81.81	82.15	145,817	82.64	82.47	82.82	0.66	0.41	0.91
50–54	142,228	82.43	82.25	82.61	155,948	82.79	82.62	82.96	0.35	0.11	0.60
55–59	138,116	84.13	83.95	84.31	136,605	83.95	83.77	84.13	− 0.18	− 0.43	0.07
60–64	139,594	85.29	85.12	85.46	136,189	85.20	85.03	85.38	− 0.09	− 0.33	0.16
65–69	150,219	86.69	86.53	86.85	138,083	86.45	86.29	86.62	− 0.23	− 0.46	0.00
70–75	116,479	84.18	83.99	84.38	133,861	85.13	84.96	85.31	0.95	0.69	1.21
Cohabitation											
Yes	639,756	88.62	88.55	88.70	639,126	88.54	88.47	88.61	− 0.08	− 0.18	0.02
No	357,050	76.93	76.81	77.05	359,585	77.39	77.27	77.51	0.46	0.29	0.63
Level of education											
Low	150,608	78.01	77.82	78.19	132,025	77.08	76.88	77.28	− 0.93	− 1.20	− 0.66
Intermediate	465,077	84.48	84.38	84.57	458,064	84.43	84.33	84.53	− 0.05	− 0.18	0.09
High	376,740	86.96	86.86	87.06	403,215	87.21	87.12	87.31	0.26	0.12	0.39
Income											
Lowest decile	85,270	71.80	71.55	72.06	85,670	72.19	71.93	72.44	0.38	0.02	0.74
Decile 2–4	289,236	81.36	81.23	81.49	290,381	81.61	81.48	81.73	0.25	0.07	0.43
Decile 5–10	622,300	87.43	87.36	87.51	622,661	87.46	87.38	87.54	0.03	− 0.08	0.13
Main source of income											
Employment/retirement	877,908	87.00	86.94	87.07	882,134	86.87	86.80	86.94	− 0.13	− 0.23	− 0.04
Student finance	3,463	75.71	74.47	76.95	3,290	76.64	75.37	77.90	0.93	− 0.85	2.70
Care of sick child/relative	3,952	68.41	67.21	69.61	4,415	72.50	71.37	73.62	4.09	2.45	5.73
Social assistance/benefits	97,949	68.77	68.52	69.01	95,447	69.67	69.42	69.91	0.90	0.56	1.24
No income	13,534	56.00	55.37	56.63	13,426	56.89	56.26	57.53	0.89	0.01	1.78
Home ownership											
Yes	774,600	87.66	87.59	87.73	773,668	87.68	87.61	87.75	0.02	− 0.08	0.12
No	205,775	73.47	73.31	73.63	213,390	74.13	73.97	74.29	0.66	0.43	0.89
Country of birth											
Sweden	861,164	85.95	85.88	86.02	845,066	86.30	86.23	86.37	0.34	0.25	0.44
Nordic country	34,017	77.64	77.25	78.03	30,975	78.56	78.16	78.97	0.92	0.36	1.48
Europe	51,696	70.82	70.49	71.15	55,945	71.68	71.36	71.99	0.86	0.41	1.32
Other	51,625	70.88	70.55	71.21	67,883	73.21	72.92	73.49	2.32	1.89	2.76
Region											
Blekinge	28,741	87.80	87.44	88.15	28,715	86.73	86.36	87.09	− 1.07	− 1.58	− 0.56
Dalarna	53,141	83.69	83.40	83.98	51,165	84.32	84.03	84.61	0.63	0.22	1.03
Gotland	11,002	81.03	80.37	81.69	11,368	82.19	81.55	82.82	1.15	0.23	2.07
Gävleborg	52,158	83.43	83.14	83.72	50,837	83.65	83.35	83.94	0.22	− 0.20	0.63
Halland	55,339	85.38	85.11	85.65	57,119	86.71	86.45	86.97	1.33	0.95	1.70
Jämtland/Härjedalen	21,969	86.15	85.73	86.57	22,963	86.01	85.59	86.42	− 0.14	− 0.74	0.45
Kalmar	43,380	84.53	84.21	84.84	42,708	84.35	84.04	84.67	− 0.17	− 0.62	0.27
Skåne	218,281	82.31	82.17	82.46	222,339	81.83	81.68	81.97	− 0.48	− 0.69	− 0.28
Värmland	51,733	85.91	85.63	86.19	51,355	85.60	85.32	85.88	− 0.31	− 0.70	0.09
Västerbotten	42,857	85.28	84.97	85.59	42,554	86.71	86.41	87.01	1.43	1.00	1.87
Västernorrland	42,277	84.00	83.68	84.32	44,425	87.13	86.84	87.42	3.13	2.69	3.56
Västmanland	46,155	85.48	85.18	85.78	40,509	85.96	85.65	86.27	0.48	0.05	0.91
Västra Götaland	285,926	83.58	83.46	83.70	292,982	84.10	83.98	84.22	0.52	0.35	0.69
Örebro	45,574	82.03	81.71	82.35	40,874	80.69	80.35	81.03	− 1.34	− 1.81	− 0.87
Type of municipality											
Large cities	240,174	81.58	81.44	81.72	250,738	81.98	81.84	82.12	0.40	0.20	0.59
Mid−sized cities	384,838	84.55	84.44	84.65	381,547	84.58	84.47	84.68	0.03	− 0.12	0.18
Smaller cities/rural areas	371,794	85.19	85.08	85.29	366,731	85.31	85.20	85.41	0.12	− 0.03	0.27
Out-of-pocket fee (2015)											
80	45,574	82.03	81.71	82.35	40,874	80.69	80.35	81.03	− 1.34	− 1.81	− 0.87
100	329,306	83.70	83.59	83.82	335,690	84.13	84.02	84.25	0.43	0.27	0.59
120	247,022	82.91	82.78	83.05	251,054	82.36	82.22	82.49	− 0.56	− 0.75	− 0.36
150	172,726	84.61	84.46	84.77	175,672	86.01	85.86	86.16	1.40	1.18	1.62
200	203,905	84.76	84.62	84.90	196,623	85.19	85.05	85.34	0.43	0.23	0.63

When assessing how the time period associated with attendance among all women in the study sample, the odds of attending were not statistically higher in 2017–2018 than in 2014–2015, neither in an unadjusted analysis (OR 1.01, 95% CI 0.99–1.02) nor when adjusting for region, age, cohabitation, education, income, main source of income, home ownership, and country of birth (OR 1.00, 95% CI 0.99–1.01).

Table 3 presents attendance before and after the fee removal in sociodemographic sub-groups at risk of low attendance. Although the change between time periods was statistically significant in all six of the main sub-groups, it was small and only exceeded one percentage point for those born in non-Nordic countries—where attendance increased by 1.7 points from 2014–2015 to 2017–2018. This effect remained statistically significant in the multivariable analysis. Among women with a low level of education, attendance decreased by 0.9 points and this effect persisted in the multivariable analysis. Attendance increased from 59.2 to 62.0% (+ 2.8 points, 95% CI 2.2–3.4) among women with a combination of any four or more risk factors for low attendance, but did not change substantially among those with three or fewer risk factors. When risk factors were combined, the groups decreased considerably in size and women with four or more risk factors constituted only 3.2% of all women invited during 2014–2015. When two specific risk factors were combined, the lowest attendance in 2014–2015 was found among women who had the lowest income and were living without a partner (53.0%) and among women whose main source of income was social assistance and benefits and either lived without a partner (60.9%) or did not own their home (61.2%). The biggest increase in attendance was found among non-Nordic women with the lowest income (+ 2.9 points, 95% CI 2.3–3.6). Other larger sub-groups where attendance increased by more than two points were women born outside of the Nordic countries who were either living alone (+ 2.1 points, 95% CI 1.6–2.6), did not own their home (+ 2.0 points, 95% CI 1.5–2.5), or whose main source of income was social assistance and benefits (+ 2.1 points, 95% CI 1.5–2.7) and women whose main source of income was social assistance and benefits and who did not own their home (+ 2.3 points, 95% CI 1.8–2.8). In all combined risk groups, the association between time period and attendance remained statistically significant in the multivariable analysis.

**Table 3:** Effect of time period on mammography screening attendance in selected sub-groups with low attendance. Mixed logistic regression analysis of attendance in 2017–2018 vs. 2014–2015

Sub-group	2014–2015		2017–2018		Change (95% CI)			Unadjusted	Adjusted
	*n*	%	*n*	%	PP	Lower CI	Upper CI	OR (95% CI)	OR (95% CI)
Risk factor for low attendance									
Living without partner (alone)	357,050	76.93	359,585	77.39	0.46	0.29	0.63	1.02 (1.01–1.03)	1.01 (0.99–1.02)^a^
Low level of education	150,608	78.01	132,025	77.08	− 0.93	− 1.20	− 0.66	0.94 (0.92–0.96)	0.95 (0.93–0.97)^b^
Lowest income	85,270	71.80	85,670	72.19	0.38	0.02	0.74	1.02 (0.99–1.04)	1.02 (1.00–1.05)^c^
Social assistance/benefits	97,949	68.77	95,447	69.67	0.90	0.56	1.24	1.05 (1.03–1.07)	1.04 (1.02–1.06)^d^
Not owning home	205,775	73.47	213,390	74.13	0.66	0.43	0.89	1.04 (1.02–1.05)	1.01 (1.00–1.03)^e^
Non-Nordic	103,321	70.85	123,828	72.51	1.66	1.34	1.97	1.08 (1.06–1.10)	1.05 (1.03–1.07)^f^
Index of the above 6 risk factors^g^									
0 risk factor	411,288	91.63	413,250	92.03	0.41	0.29	0.52	1.12 (1.08–1.15)	1.11 (1.07–1.15)
1	313,432	86.11	311,851	86.11	0.00	− 0.16	0.16	0.98 (0.95–1.01)	0.97 (0.93–1.00)
2	176,436	78.15	173,189	77.95	− 0.20	− 0.44	0.05	0.98 (0.97–1.00)	0.98 (0.96–1.00)
3	65,709	66.04	66,390	66.83	0.79	0.37	1.20	1.03 (1.01–1.06)	1.03 (1.01–1.06)
4	22,675	58.61	24,727	61.32	2.71	2.03	3.39	1.12 (1.08–1.16)	1.13 (1.10–1.18)
5	8,116	60.89	9,398	63.71	2.82	1.69	3.96	1.19 (1.10–1.28)	1.23 (1.14–1.33)
6	877	59.14	1,108	62.32	3.18	− 0.19	6.55	1.20 (0.97–1.49)	1.22 (0.98–1.52)
4 or more	31,668	59.19	35,233	61.97	2.78	2.20	3.36	1.12 (1.09–1.16)	1.14 (1.10–1.17)
5 or 6	8,993	60.71	10,506	63.56	2.85	1.77	3.92	1.13 (1.05–1.21)	1.21 (1.13–1.30)
Selected combinations^h^									
Lowest income + living alone	15,854	53.02	16,989	54.81	1.79	1.00	2.58	1.07 (1.03–1.11)	1.07 (1.02–1.12)^i^
Lowest income + social assist	18,462	64.83	20,734	67.33	2.50	1.73	3.26	1.17 (1.12–1.22)	1.15 (1.09–1.21)^j^
Lowest income + not owning	24,956	64.28	27,945	66.26	1.98	1.32	2.63	1.09 (1.06–1.13)	1.10 (1.05–1.14)^k^
Non-Nordic + lowest income	26,128	62.87	31,038	65.79	2.92	2.29	3.55	1.14 (1.10–1.17)	1.17 (1.12–1.21)^l^
Non-Nordic + living alone	39,079	64.13	47,236	66.21	2.09	1.57	2.60	1.09 (1.06–1.12)	1.06 (1.02–1.09)^m^
Non-Nordic + social assist	26,803	66.85	29,459	68.96	2.11	1.47	2.75	1.10 (1.06–1.14)	1.13 (1.08–1.17)^n^
Non-Nordic + not owning	44,735	66.56	54,933	68.58	2.02	1.54	2.50	1.10 (1.07–1.13)	1.07 (1.04–1.10)^o^
Social assist + living alone	48,165	60.86	48,068	62.60	1.74	1.26	2.22	1.08 (1.05–1.11)	1.07 (1.04–1.10)^p^
Social assist + not owning	41,403	61.19	42,936	63.51	2.32	1.80	2.83	1.11 (1.08–1.14)	1.08 (1.05–1.11)^q^

*ORs* Odds Ratios, *CIs* 95% confidence intervals, *n* number of attenders, *PP* percentage points

^a^Estimates were adjusted for region, age group, education, income, main source of income, home ownership, and country of birth

^b^Estimates were adjusted for region, age group, cohabitation, income, main source of income, home ownership, and country of birth

^c^Estimates were adjusted for region, age group, cohabitation, education, main source of income, home ownership, and country of birth

^d^Estimates were adjusted for region, age group, cohabitation, education, income, home ownership, and country of birth

^e^Estimates were adjusted for region, age group, cohabitation, education, income, main source of income, and country of birth

^f^Estimates were adjusted for region, age group, cohabitation, education, income, main source of income, and home ownership

^g^Estimates were adjusted for region and age group

^h^A combination of two risk factors was selected for inclusion where the change exceeded one percentage point and the change in the combined group exceeded the change observed for either individual risk factor. Estimates were adjusted for region, age group, and risk variables not selected for respective subgroup

^i^Estimates were adjusted for region, age group, education, main source of income, home ownership, and country of birth

^j^Estimates were adjusted for region, age group, cohabitation, education, home ownership, and country of birth

^k^Estimates were adjusted for region, age group, cohabitation, education, main source of income, and country of birth

^l^Estimates were adjusted for region, age group, cohabitation, education, main source of income, and home ownership

^m^Estimates were adjusted for region, age group, education, income, main source of income, and home ownership

^n^Estimates were adjusted for region, age group, cohabitation, education, income, and home ownership

^o^Estimates were adjusted for region, age group, cohabitation, education, income, and main source of income

^p^Estimates were adjusted for region, age group, education, income, home ownership, and country of birth

^q^Estimates were adjusted for region, age group, cohabitation, education, income, and country of birth

## Discussion

In this longitudinal population-based register study of mammography screening attendance in Sweden, we found that overall attendance remained relatively stable at 84% after removal of the out-of-pocket fee in 2016, but rose by 2.9 percentage points among non-Nordic women with the lowest income (from 62.9 to 65.8%) and by 2.8 points among women with four or more risk factors for low attendance (from 59.2 to 62.0%).

To our knowledge, no other European studies have reported on the effect of removing the fee for mammography screening on screening attendance, except for a study in Stockholm showing an increase of two percentage points after removing the out-of-pocket fee in 2012 [15]. However, a study in Finland found that the likelihood of screening attendance decreased after a fee was introduced, independently of socioeconomic status [20]. A Swedish randomized study examining the effect of offering free cervical cancer screening exams to women in socioeconomically disadvantaged areas failed to establish a statistically significant effect on attendance [21].

Although some US research has demonstrated a positive effect of reducing or removing the out-of-pocket fee on screening attendance [12–14], more recent studies investigating the effect of cost-share removal among Medicare beneficiaries show conflicting results. Some studies found no improvement in uptake in general [22–24], nor in low socioeconomic groups [23, 24], whereas others found an increase in general attendance [25, 26], as well as among unmarried women [25], but not in areas with lower education [26]. Yet another study detected a smaller decrease in attendance in the intervention group compared to a control group, but found no difference by neighborhood socioeconomic status [27].

The lack of change in attendance in the present study is somewhat surprising, considering that the odds of viewing the screening exam as too expensive were threefold among non-attenders compared to attenders according to a previous Swedish cross-sectional study [28]. However, such an opinion, no matter how strong, is just one of many factors and may not ultimately be the deciding one. Other rationales and life circumstances play into the complex decision-making process that some qualitative research studies have depicted [29, 30]. The actual out-of-pocket fee was fairly modest, and there could be other inconveniences and costs that influence the decision to attend and remain obstacles to attendance, especially among socioeconomically vulnerable groups. Such obstacles might include transportation to the screening site, making the effort to reschedule, and taking time off from work [31]. This may explain why only removing the out-of-pocket cost does not appear sufficient to impact uptake in these groups.

### Strengths and limitations

Removal of the screening fee was implemented simultaneously in all health care regions included in this study. However, the absence of control groups or a staggered design, and the lack of means to adequately control for other changes and interventions that may have been introduced in different regions during the study period, limit our ability to evaluate a causal effect of introducing free mammography screening on screening attendance. Another requirement for establishing a causal relationship is evidence of a dose–response relationship, which our results do not demonstrate. Although no change in average attendance was noticeable among regions charging 80–120 SEK, a larger average increase occurred among regions that charged 150 SEK than among those charging 200. An absence of a correlation between the fee and the attendance is corroborated by a Swedish study examining mammography screening attendance in 1995–1996 [4].

This study assesses screening attendance up to and including 2018, i.e., two years after eliminating the screening fee, which may not have been a sufficient amount of time for change to occur. There was no standardized process of informing women about the fee removal across regions—although a survey among the regions indicated that most of them at least included information about the screening exam being free of charge in the letter of invitation—and the level of awareness of this change in the study population is unknown. Such awareness is a necessary condition for establishing a causal effect on attendance.

The screening attendance, based on the actual appointment date within each time period 2014–2015 and 2017–2018, that is presented in this study, might be somewhat higher than screening attendance based on the primary appointment date specified in the invitation. In about 45% of the cases, these dates were the same (based on data for 2017 and 2018), but a majority of the primary appointment dates were rescheduled. For 2017 and 2018, we had relatively complete data for the primary appointment date, but not for 2014 and 2015. For comparative reasons, we had to use the same definition of attendance for both time periods. Among those who were invited to be screened during 2017–2018, 82.8% attended within 90 days of the primary appointment, which is 1.3 percentage points lower than the attendance rate that we present. However, we believe that this difference would have been similar in size for the time period 2014–2015.

Despite these limitations, our population study has many strengths, including its large size, covering the majority of Sweden’s regional mammography programs. While many studies may struggle with low response rates and selection bias and rely on self-reported data for both exposure and outcome measures, we obtained this information from high-quality register data, originating from several different official sources with high coverage [16], thus minimizing measurement error and misclassification.

## Conclusion

Screening attendance did not undergo any important increase after implementing free screening, although attendance among some sociodemographic groups increased by almost three percentage points after the policy change. These findings may apply to similar population-based screening programs in countries with universal health care, high screening uptake, and small out-of-pocket fees.

## Data Availability

The data can be made available upon reasonable request.

## Abbreviations

OR: Odds ratio
CI: Confidence interval
SEK: Swedish kronor (currency)
